# Preparation of Dräger Atlan A350 and General Electric Healthcare Carestation 650 anesthesia workstations for malignant hyperthermia susceptible patients

**DOI:** 10.1186/s12871-021-01533-0

**Published:** 2021-12-13

**Authors:** Sebastian Heiderich, Christian Thoben, Nils Dennhardt, Terence Krauß, Robert Sümpelmann, Stefan Zimmermann, Michael Reitz, Henrik Rüffert

**Affiliations:** 1 Clinic of Anaesthesiology and Intensive Care Medicine, Hannover Medical School, Carl-Neuberg-Str. 1, 30625 Hannover, Germany; 2 Department of Sensors and Measurement Technology, Leibniz University Hannover, Institute of Electrical Engineering and Measurement Technology, Hannover, Germany; 3 Clinic of Anaesthesiology and Intensive Care Medicine, Helios Klinik Schkeuditz, Leipzig, Germany

**Keywords:** Malignant hyperthermia, Trigger-free anesthesia, Patient safety, Volatile anesthetics, Succinylcholine

## Abstract

**Background:**

Patients at risk of malignant hyperthermia need trigger-free anesthesia. Therefore, anesthesia machines prepared for safe use in predisposed patients should be free of volatile anesthetics. The washout time depends on the composition of rubber and plastic in the anesthesia machine. Therefore, new anesthesia machines should be evaluated regarding the safe preparation for trigger-free anesthesia. This study investigates wash out procedures of volatile anesthetics for two new anesthetic workstations: Dräger Atlan A350 and General Electric Healthcare (GE) Carestation 650 and compare it with preparation using activated charcoal filters (ACF).

**Methods:**

A Dräger Atlan and a Carestation 650 were contaminated with 4% sevoflurane for 90 min. The machines were decontaminated with method (M1): using ACF, method 2 (M2): a wash out method that included exchange of internal parts, breathing circuits and soda lime canister followed by ventilating a test lung using a preliminary protocol provided by Dräger or method 3 (M3): a universal wash out instruction of GE, method 4 (M4): M3 plus exchange of breathing system and bellows. Decontamination was followed by a simulated trigger-free ventilation. All experiments were repeated with 8% desflurane contaminated machines. Volatile anesthetics were detected with a closed gas loop high-resolution ion mobility spectrometer with gas chromatographic pre-separation attached to the bacterial filter of the breathing circuits. Primary outcome was time until < 5 ppm of volatile anesthetics and total preparation time.

**Results:**

Time to < 5 ppm for the Atlan was 17 min (desflurane) and 50 min (sevoflurane), wash out continued for a total of 60 min according to protocol resulting in a total preparation time of 96-122 min. The Carestation needed 66 min (desflurane) and 24 min (sevoflurane) which could be abbreviated to 24 min (desflurane) if breathing system and bellows were changed. Total preparation time was 30-73 min. When using active charcoal filters time to < 5 ppm was 0 min for both machines, and total preparation time < 5 min.

**Conclusion:**

Both wash out protocols resulted in a significant reduction of trace gas concentrations. However, due to the complexity of the protocols and prolonged total preparation time, feasibility in clinical practice remains questionable. Especially when time is limited preparation of the anesthetic machines using ACF remain superior.

**Supplementary Information:**

The online version contains supplementary material available at 10.1186/s12871-021-01533-0.

## Introduction

Malignant hyperthermia (MH) is an inherited pharmaco-genetic disorder in which affected individuals are at risk to develop life-threatening metabolic crises when in contact to volatile anesthetics and/or succinylcholine. Whenever patients with a known susceptibility to malignant hyperthermia (MHS), or patients with MH associated myopathies (central core disease, King-Denborough syndrome and some other rare muscle disorders) need anesthesia, either regional or so called “trigger-free” general anesthesia is recommended. This includes, that the anesthetic machine should be clean of residual volatile anesthetics. Unfortunately, cleaning modern anesthetic machines highly differs from manufacturer and device because volatile anesthetics adsorb and desorb to different amount of rubber and plastic components of the anesthetic machine [1]. The European Malignant Hyperthermia Group (EMHG) have developed consensus guidelines on perioperative management of MHS patients that contains recommendations on the elimination of residual trace concentrations of volatile anesthetics in the machine to ensure a trigger-free anesthesia [2]. Basically, three different approaches are recommended: First: Using a spare anesthetic machine which had never contact to any volatile anesthetic. Second: Using activated charcoal filters (ACF) to eliminate trace gas concentrations. Third: Preparing the anesthetic machines by washing out volatile anesthetics according to manufacturer’s instructions of the device. The first two approaches can be less cost effective depending on the circumstances (operation room workload and estimated number of trigger-free anesthesia per year) [3]. Regarding the wash out method, most manufacturer does not give approved instructions how to wash out the volatile anesthetics from the anesthetic machines. In the literature most anesthetic machines are evaluated regarding the preparation process for trigger-free anesthesia [1, 3–5]. The EMHG guidelines provide an overview of the known wash out times for most anesthesia machines, but data is missing for the newest generation devices. Therefore, this study aims to investigate wash out profiles of the Dräger Atlan 350 and General Electric Healthcare (GE) Carestation 650. There are no official instructions available for both machines yet. However, we used a preliminary wash out protocol from Dräger and a general instruction from GE in which the steps are standardized for all devices varying only by the wash out time needed for each machine series [6]. This study should help clinicians to decide which approach to trigger-free anesthesia is most practicable and economic for their individual hospital.

## Methods

### Contamination phase

A test lung was ventilated with 8% desflurane or 4% sevoflurane for 90 min using the Autoflow Mode (AF) of the Atlan A350, and Pressure Control Ventilation-Volume Guaranteed (PCV-VG) of the Carestation 650. Other settings were as following: FGF of 4 l⋅min^− 1^, tidal volume 500 mL, respiratory rate 12 breaths/minute, positive end-expiratory pressure (PEEP) 5 mbar, inspired to expired time ratio of 1:1.9 for the Atlan A350 and 1:2 for the Carestation 650. The high concentrations of volatile anesthetics were chosen to create rather heavily contaminated machines with the intention to set up a worst-case scenario. Therefore, the anesthetic machines were contaminated with the same contamination method as previously reported [7].

### Preparation methods

After contamination phase different methods were used to clean the machines from volatile anesthetics. Each method was tested separately on sevoflurane and desflurane contaminated machines. The preparation methods are shown more in detail in Table 1. The preparation was conducted together by two anesthetic consultants (authors S. H and T.K.).

**Table 1:** Experimental Setup

	Method 1	Method 2	Method 3	Method 4
Workstation	Atlan A350	Both workstations	Carestation 650	Carestation 650
Procedure origin	Dräger (draft)	Dynasthetics (ACF instruction manual)	GE (technical report)	GE (technical report plus breathing system and bellows changed)
1	Standby mode	Vaporizer removed	Vaporizer removed	Vaporizer removed
2	Vaporizer removed	Manual ventilation with 10 l⋅min^− 1^ FGF for 90 s	Breathing circuits, breathing bag, bacterial filter and CO_2_ sampling line changed.	Breathing circuits, breathing bag, bacterial filter and CO_2_ sampling line changed
3	Breathing circuits, breathing bag and CO_2_ sampling line removed	ACF attached to both inspiratory and expiratory limps	Washout ventilation: O_2_ = 100%, FGF = 15 l⋅min^− 1^, frequency = 12⋅min^− 1^, VT = 700 ml, I:E = 1:2, PEEP = off.	sodium lime canister, water trap, breathing system and bellow changed.
4	Sodium lime canister and adapter removed	Breathing circuits, breathing bag, bacterial filter and CO_2_ sampling line changed	Ventilation stopped when concentration of volatile anaesthetics dropped to < 5 ppm.	Washout ventilation: O_2_ = 100%, FGF = 15 l⋅min^− 1^, frequency = 12⋅min^− 1^, VT = 700 ml, I:E = 1:2, PEEP = off.
5	Piston diaphragm and breathing system changed	System leak test	Bag-vent switch to vent position	Ventilation stopped when concentration of volatile anaesthetics dropped to < 5 ppm.
6	New sodium lime adapter without canister attached	Test lung ventilated with 10 l⋅min^− 1^ FGF	breathing circuit, breathing bag, sodium lime canister, bacterial filter and CO_2_ sampling line changed.	Bag-vent switch to vent position
7	30 s O_2_-flush		System leak test	breathing circuit, breathing bag, sodium lime canister, water trap, bacterial filter and CO_2_ sampling line changed.
8	New breathing circuits, breathing bag and CO_2_ sampling line attached		10 s O_2_-flush	System leak test
9	System leak test		Test lung ventilated with 15 l⋅min^− 1^ FGF	10 s O_2_-flush
10	APL-valve to 30cmH_2_O			Test lung ventilated with 15 l⋅min^− 1^ FGF
11	Test lung attached			
12	Breathing bag removed			
13	Sodium lime adapter removed			
14	60 min washout: ventilation mode = volume controlled, O_2_ = 100%, FGF 15 l⋅min^− 1^, frequency = 40⋅min^− 1^, Pmax = 70cmH_2_O, VT = 900 ml, PEEP = off, Tinsp = 0.7 s, Tplat = 20%			
15	Standby mode			
16	Piston diaphragm changed			
17	Breathing circuits, breathing bag and CO_2_ sampling line removed, test lung			
18	New sodium lime adapter and canister attached			
19	30 s O_2_-flush			
20	New breathing circuits, breathing bag and CO_2_ sampling line attached			
21	System leak test			
22	Breathing bag removed ventilation mode = Man/Spon APL-valve to 30cmH_2_O FGF = 4 l⋅min^−1^ wait for “patient”			
23	FGF = 10 l⋅min^− 1^ 30 s O_2_-flush breathing bag re-attached attach bacterial filter			
24	Test lung ventilated with a FGF of 12 l⋅min^1^			

Wash out procedures used in this study. *ACF* Activated charcoal filter, *FGF* Fresh gas flow, *GE* General Electric Healthcare, *APL* Adjustable pressure-limiting, *VT* Tidal volume, *Pmax* Maximum pressure, *Tinsp* Inspiratory time, *Tplat* Time of plateau pressure

### Active charcoal filter procedure

Method 1 (M1): Both anesthetic machines were also prepared using ACF: The Vaporizer was turned off, manual mode was selected, APL ventil turned to zero (Spont), a FGF of 10 l⋅min^− 1^ was selected for 90 s. After that, activated charcoal filters were inserted to both the inspiratory and expiratory limb. Subsequently, breathing circuits, breathing bag, bacterial filter, CO_2_ sampling line, sodium lime canister and test lung was changed. This procedure was taken from the manufacturer instructions (https://www.dynasthetics.com/Vapor-Clean/Vapor-Clean-IFU.pdf). M1 was performed four times: on a sevoflurane contaminated Atlan A350, desflurane contaminated Atlan A350, sevoflurane contaminated Carestation 650 and desflurane contaminated Carestation 650.

### Atlan A350 procedure

Method 2 (M2): A preliminary draft protocol of the Dräger company was used to clean the machine: This included vapor removal, change of breathing circuits, breathing bag, bacterial filter, CO_2_ sampling line, sodium lime canister, water trap, breathing system and breathing membrane. The protocol included a 60 min ventilation of a test lung at a FGF of 15 l⋅min^− 1^.

M2 was performed on one sevoflurane contaminated machine and one desflurane contaminated machine.

### Carestation 650 procedure

Method 3 (M3): Preparation according to universal manufacturer’s instructions [6]. First, the vaporizer was removed. Then breathing circuits, breathing bag, bacterial filter, CO_2_ sampling line and test lung was changed. A system leak test was performed. After that, the test lung was ventilated using PCV-VG Mode, FGF 15 l⋅min^− 1^, tidal volume 700 mL, respiratory rate of 12 breaths/minute, inspired to expired time ratio of 1:2 with no PEEP. As no data on the washout time is available for the Carestation 650, the ventilation was continued until the volatile anesthetic dropped to < 5 ppm. After that, the bag-vent switch set to vent position. After that breathing circuits, breathing bag, bacterial filter, CO_2_ sampling line, sodium lime canister and test lung was changed. The O_2_ flush was activated for 10 s. After that the preparation was finished and the experiment continued with “waiting for the simulated patient”. M3 was performed on one sevoflurane and one desflurane contaminated machine.

Method 4 (M4): Like M3 plus an additional change of breathing system and bellows with autoclaved spare parts at the beginning of the preparation. Also, the water trap and soda lime canister both were changed twice: in the beginning and at the end of the preparation.

A preparation with active charcoal filters was also carried out for the Carestation (see M2). M4 was performed on one sevoflurane and one desflurane contaminated machine.

### Waiting for the simulated patient and trigger-free ventilation

After the preparation process a simulated waiting time for the patient was set to 20 min where the machines were set to manual mode with a FGF of 4 l⋅min^− 1^. Then, a new test lung was ventilated for 20 min using the same ventilation settings as during the contamination phase.

The FGF during the simulation was chosen in accordance with manufacturer’s instructions:

Dynasthetics: M1: 3 l⋅min^− 1^.

Dräger: M2: 12 l⋅min^− 1^ (double minute volume).

GE: M3, M4: 15 l⋅min^− 1^.

After that, a rebound phenomenon was provoked: In experiments M1 the ACF were removed, and ventilation was continued with 3 l⋅min^− 1^ FGF. In experiments M2, M3 and M4 ventilation was continued using a reduced FGF of 1 l⋅min^− 1^. This was chosen to reveal residual contamination of the machines. It is not recommended to do this in clinical practice.

### Main outcome measures

For both anesthetic machines, the washout time of desflurane and sevoflurane was measured. It was defined as the time needed from start of the test-lung ventilation to the reduction of inspiratory volatile anesthetic gas concentration < 5 ppm. Also, the total preparation time for each method was logged. It was defined as the time needed for the whole procedure from the beginning of the preparation until the machine was ready for trigger-free ventilation, that included assembling and dissembling of the equipment.

### Ion mobility spectrometry

In all experiments, a high-resolution (R_P_ = 90) ion mobility spectrometer (IMS) with gas chromatographic pre-separation (GC) operated with a closed gas loop is used to determine the concentrations of volatile anesthetics. The GC-IMS was developed by Leibniz University Hannover. In ion mobility spectrometry analyte molecules are first ionized and subsequently identified by their ion mobility in a so-called drift gas under the influence of an electric field. IMS provide highest sensitivity and detection limits in the low parts per billion (ppb) and even parts per trillion (ppt) range in less than a second of measuring time. Today, even highest resolution can be reached [8]. However, in this study the measured concentrations are far above the detection limit. Brief details relevant to this study are given here; a detailed description of the system and its applications can be found elsewhere [9–11]. A 10 m standard capillary column (Restek, RTX volatiles, ID 530 μm, film thickness 2 μm) at a constant temperature of T = 50 °C is used as GC column. The sample loop volume is just 10 μl, due to the high sensitivity of the IMS and the high concentrations of the volatile anesthetics. During operation, the sample loop is flushed by a defined gas flow of 50 ml⋅min^− 1^ from the sample inlet which was connected to the bacterial filter via a gas sample line. The sample loop volume is injected via a 6-port-valve into the GC carrier gas stream with a carrier gas flow of 5 ml⋅min^− 1^, resulting in a GC run time of 60 s. Thus, all peaks found in GC-IMS measurements are characterized by their ion mobility, GC retention time and peak area, which relates to the compound concentration. The GC-IMS was calibrated with homemade permeation tubes using a Vici Dynacalibrator Model 150 permeation oven.

## Results

### Atlan A350

**Fig. 1:**
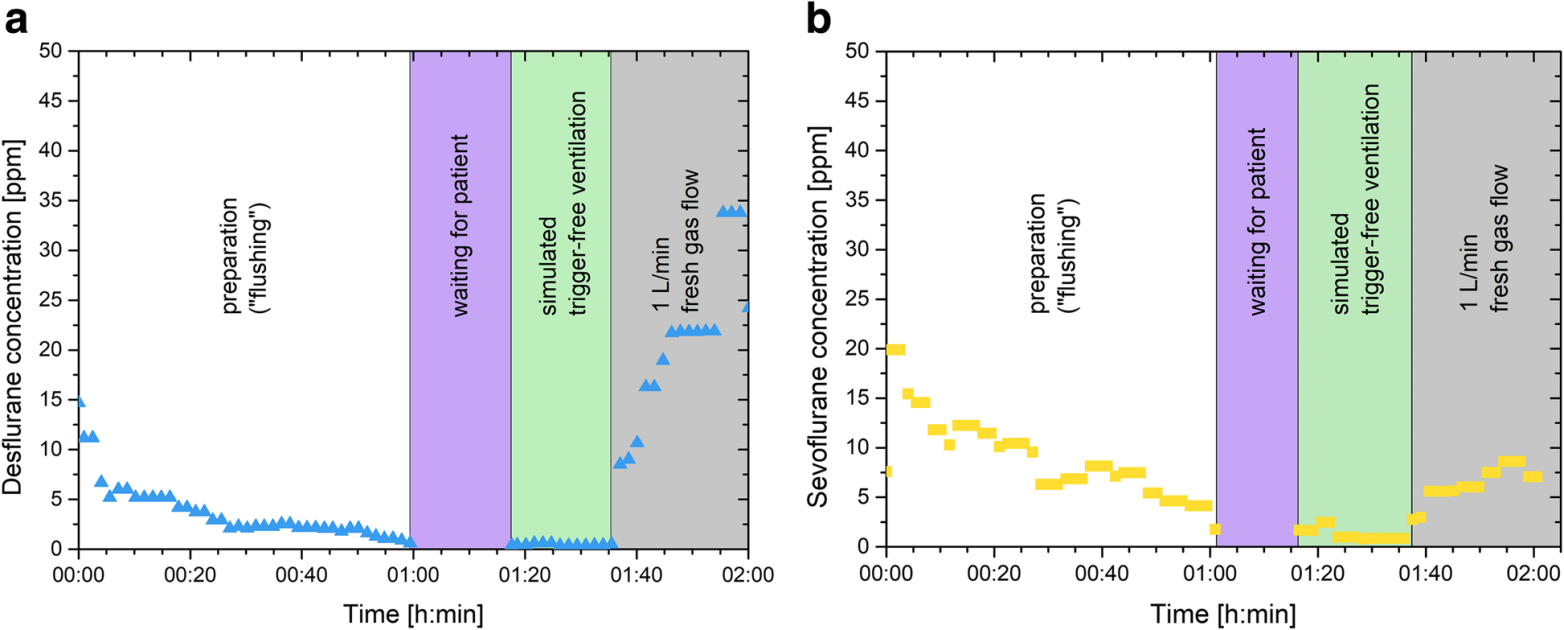
Wash out procedure and simulated trigger-free ventilation of the Atlan A350 using manufacturer’s protocol. Ppm; parts per million. During waiting time for the simulated patient, the machine was set to manual mode with a fresh gas flow of 4 l⋅min^− 1^. **A** Desflurane experiment **B** Sevoflurane experiment

When using ACF (M1) the concentrations of volatile anesthetics immediately dropped to < 1 ppm for desflurane and < 2.5 ppm for sevoflurane and remained there during simulated trigger-free ventilation until the ACF were removed: A rebound of up to 80 ppm desflurane and 50 ppm sevoflurane was observed. The wash out time of M2 was 17 min in the desflurane experiment and 50 min in the sevoflurane experiment. In both experiments wash out ventilation was continued for a total of 60 min as stated in the procedure protocol (see Table 1). During simulated trigger-free ventilation the concentrations remained below 5 ppm in both the sevoflurane and desflurane experiments. A rebound effect was observed after the FGF was reduced to 1 l⋅min^− 1^ (see Fig. 1a, b). Total preparation time, which included assembling and dissembling of the equipment, was < 5 min for M1 and 96–122 min for M2.

### Carestation 650

**Fig. 2:**
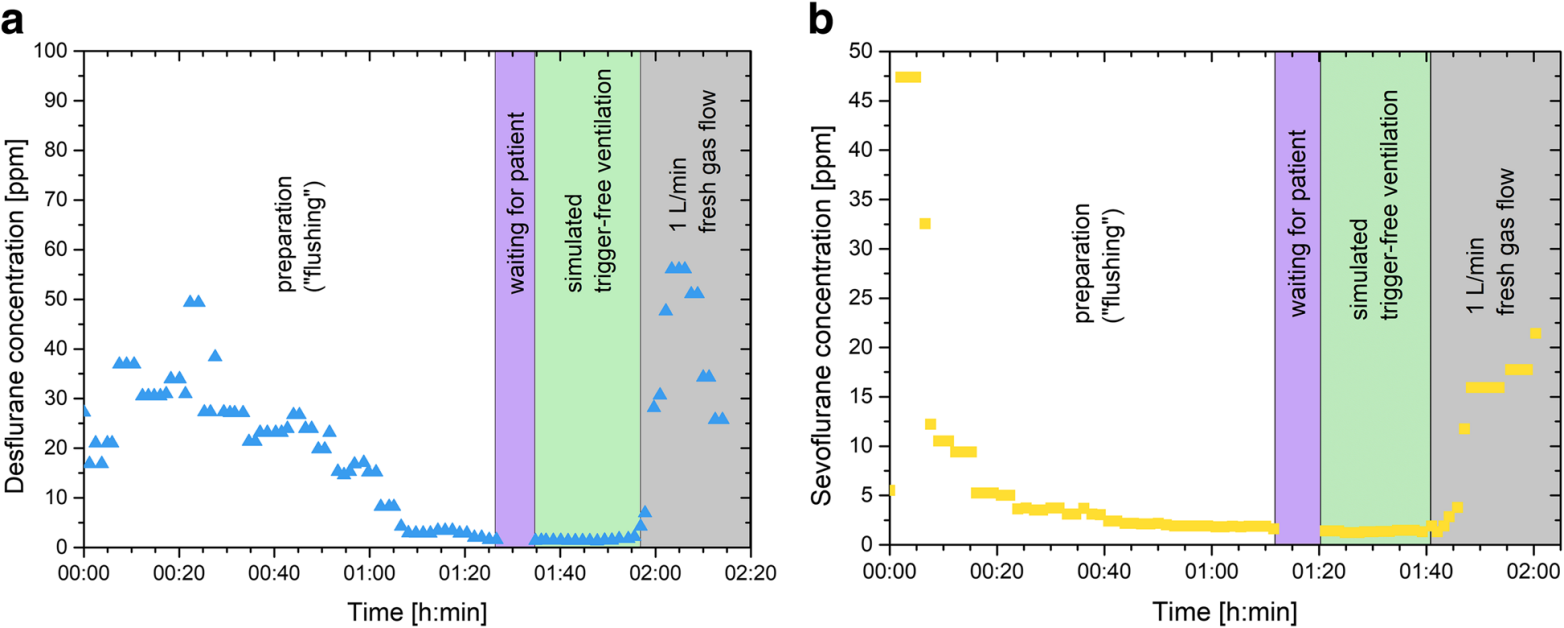
Wash out procedure and simulated trigger-free ventilation of the Carestation 650 using manufacturer’s protocol. Ppm; parts per million. During waiting time for the simulated patient, the machine was set to manual mode with a fresh gas flow of 4 l⋅min^− 1^. **A** Desflurane experiment **B** Sevoflurane experiment

**Fig. 3:**
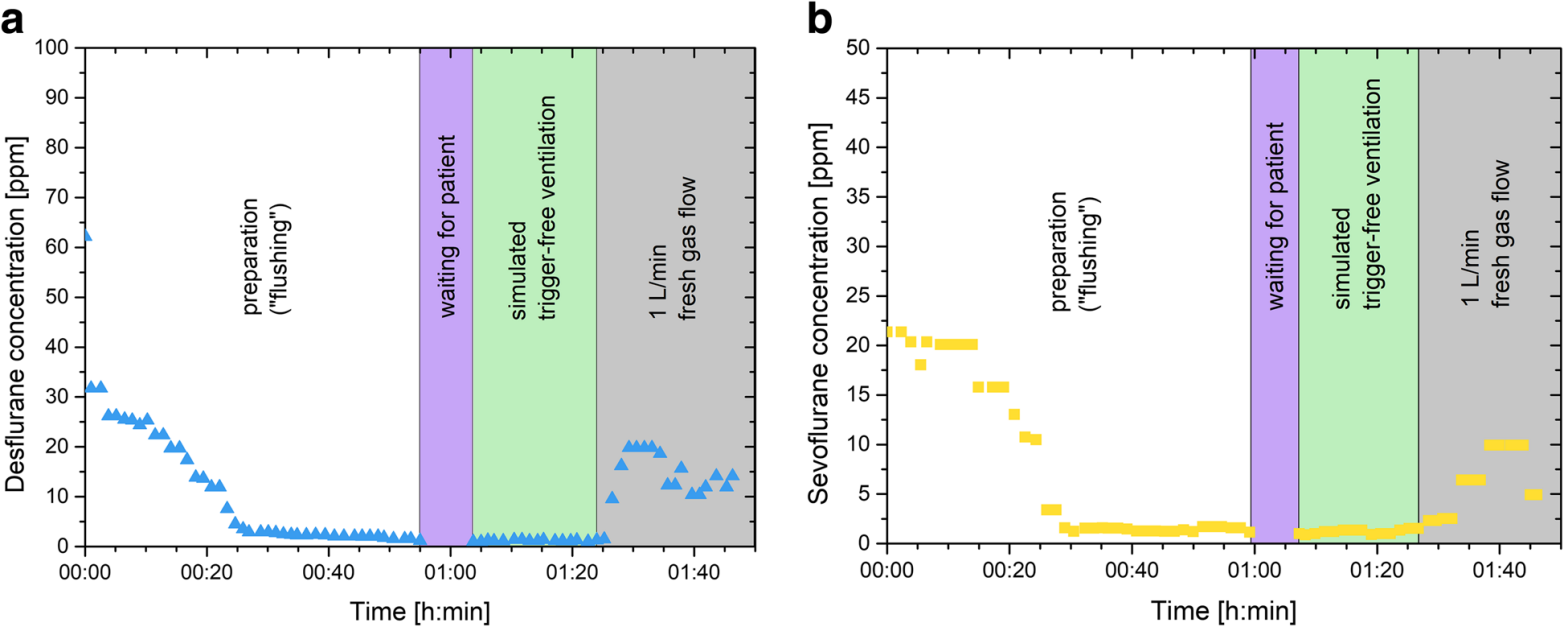
Wash out procedure and simulated trigger-free ventilation of the Carestation 650 using manufacturer’s protocol with surplus exchange of the breathing system and bellows. Ppm; parts per million. During waiting time for the simulated patient, the machine was set to manual mode with a fresh gas flow of 4 l⋅min^− 1^. **A** Desflurane experiment **B** Sevoflurane experiment

When using ACF (M1) the concentrations of volatile anesthetics immediately dropped to < 1 ppm for desflurane and < 2 ppm for sevoflurane and remained there during simulated trigger-free ventilation until the ACF were removed: A rebound of up to 25 ppm desflurane and 73 ppm sevoflurane was observed. The wash out time of M3 was 66 min in the desflurane experiment and 24 min in the sevoflurane experiment. Wash out ventilation was continued for a total of 72 and 86 min as no live information about the exact concentration was available with our detecting method used. During simulated trigger-free ventilation the concentrations remained below 5 ppm in both the sevoflurane and desflurane experiments. A rebound effect was observed after the FGF was reduced to 1 l⋅min^− 1^ (See Fig. 2a, b). In M4 the concentrations dropped after 24 min to < 5 ppm in both the sevoflurane and desflurane experiment. Wash out ventilation was continued for a total of 55 and 59 min as no live information about the exact concentration was available with our detecting method used. Rebound effects of 10 ppm sevoflurane and 34 ppm desflurane were recorded after reducing the FGF to 1 l⋅min^− 1^ (See Fig. 3a, b). Total preparation time was < 5 min (M1), 30–73 min (M3) and 31–37 min (M4).

## Discussion

### Wash out time is not equal to preparation time

The washout time of the Atlan A350 (M2) was < 60 min and therefore shorter than similar products of the same manufacturer and comparable other anesthetic machines [1]. This might be due to the extensive protocol used in M2: all exchangeable parts were changed, including the breathing system. The breathing circuits and piston diaphragm were changed even twice. Unfortunately, this led to an extensive overall preparation time (96–122 min in this study) which would surely benefit from a training effect. Still, trigger-free ventilation is a rare need, and therefore any routine experience of the nurses and anesthesiologists may remain limited. Also, the vast number of steps in this protocol (see Table 1) may lead to non-compliance or mistakes and therefore seems not suitable in clinical practice. The desflurane washout time of the Carestation 650 (M3) was longer than other anesthetic machines from GE as described in their technical report [6]. This highlights the need to gain experimental data from every new machine. However, the prolonged desflurane wash out time was shortened when the breathing system and bellows were changed (M4). This could be considered a feasible alternative method to clinicians.

### Active charcoal filters vs. wash out procedures

Both the EMHG and the Malignant Hyperthermia Association of the United States (MHAUS) recommend using ACF (Vapor-Clean™, Dynasthetics, Salt Lake City, UT) as alternative method to prepare anesthetic machines for trigger-free anesthesia. The preparation with ACF (M1) was – not surprisingly – more user-friendly and less time consuming. Moreover, the absolute concentrations of volatile anesthetics were lower (< 1 ppm) compared to the washout protocols of both machines. The advantage of the ACF lies in the simplicity (short and clear instruction checklist), speed (readiness within 5 min) and universality for all different anesthetic machines. Therefore, in case of emergency surgery or reorganization of the operation schedule the ACF seems more practicable. The only disadvantage is the extra material costs (85€ in Germany). Still, in some circumstances (operation schedules with high workload) the use of ACF can be more cost efficient compared to wash out procedures when taking loss of operation time into account [3].

### Rebounds and fresh gas flow

All experiments showed a relevant rebound effect after reduction of FGF, or removal of ACF respectively. This shows that both anesthetic machines are continuing to emit volatile anesthetics. Therefore, even after the washout procedure the machines must be considered as contaminated and any reduction of FGF or even short standby time should be avoided during trigger-free ventilation. This may be problematic in pediatric anesthesia, were a low FGF is recommended to avoid loss of heat and moisture. If using ACF, the filters should be remained in place for the whole anesthetic procedure [2]. The manufacturer recommend changing ACF after 12 h, but in previews studies we demonstrated a safe use of the ACF for 24 h [7].

### Limitations of the study

#### Repetition of the experiments

In this study all experiments were only performed once per method, machine, and anesthetic gas. Different machines of the same type with different levels of contamination may result in different wash out time needed to get below 5 ppm.

#### Hardware and software versions

Deviations of the product series, software version and equipment may potentially affect the washout times of volatile anesthetics. The experiments were conducted using new machines previously used for demonstration on exhibitions. In clinical practice, anesthetic machines may show different levels of contamination after years of repetitive exposure to volatile anesthetics. Therefore, a rather high contamination (4% sevoflurane and 8% desflurane) was chosen. Thereby, measurements were taken in a worst-case scenario to avoid false low wash out times.

#### Material aging and contact time

Both anesthetic machines include internal unchangeable rubber and plastic components. The material characteristics may change over the years. Plastic and rubber are known to become more porous over time which may lead to a different adsorption and desorption rates of volatile anesthetics. Therefore, the wash out times needed for old machines of the same type may differ from this experiment.

#### Ventilation modes

Both anesthetic machines are overly complex and the gas flow directions and speed inside the machines are depending on the ventilation mode in use. Therefore, conclusions may only be drawn in respect to the ventilation modes used in this study.

## Conclusion

Current guidelines from EMGH and MHAUS recommending a concentration of < 5 ppm of any volatile anesthetic during trigger-free anesthesia. If choosing a washout procedure to reach that goal, manufacturer’s instructions should be used if available. The procedures are more complex than a simple flush for modern anesthetic machines. Therefore, total preparation time is longer than the washout time itself. This study showed that a preliminary washout procedure of Dräger meet the requirements of trigger-free anesthesia for the Atlan A350 in this study. Even though the time to < 5 ppm were only 17 min (desflurane) and 50 min (sevoflurane) we still recommend conducting the full 60 min of wash out ventilation of the protocol, because contamination of the machines may vary as stated in our limitation section. In this study the general instructions of GE [6] were successfully used to prepare the Carestation 650. Here, the wash out times were 24 min (sevoflurane) and 66 min (desflurane), which could be shortened with an exchange of the breathing system and bellows to 24 min. Because of the prolonged total preparation time in both machines and the complexity of the procedures the use of ACF is more feasible in clinical practice especially when time is critical. Future studies should further investigate rebound effects during trigger-free anesthesia, because in clinical practice ventilation modes and FGF rates are frequently changed during general anesthesia.

## Supplementary Information

**Additional file 1.**

## Data Availability

The datasets used and/or analyzed during the current study are available from the corresponding author on reasonable request.

## Abbreviations

ACF: Activated charcoal filters
AF: Autoflow Mode
APL: Adjustable pressure-limiting
EMHG: European Malignant Hyperthermia Group
FGF: Fresh gas flow
GC: Gas chromatographic pre-separation
GE: General Electric Healthcare
IMS: Ion mobility spectrometer
M1: Method 1
M2: Method 2
M3: Method 3
M4: Method 4
MAC: Minimum alveolar concentration
MH: Malignant hyperthermia
MHS: Malignant hyperthermia susceptible
MHAUS: Malignant Hyperthermia Association of the United States
PCV-VG: Pressure control ventilation-volume guaranteed
Pmax: Maximum pressure
ppb: Parts per billion
ppt: Parts per trillion
Tinsp: Inspiratory time
Tplat: Time of plateau pressure
VT: Tidal volume
