# Supplementary material for: Preparation of Dräger Atlan A350 and General Electric Healthcare Carestation 650 anesthesia workstations for malignant hyperthermia susceptible patients
Source: BMC Anesthesiol. 2021 Dec 13;21:315. doi: 10.1186/s12871-021-01533-0 (PMC8667359; doi:10.1186/s12871-021-01533-0)
Supplement: Supplementary file 1 — Additional file 1. [file 12871_2021_1533_MOESM1_ESM.docx]

Supplemental Table: Materials used in this study

| Item | Dräger  Atlan A350 | General Electric Healthcare Carestation 650 |
| --- | --- | --- |
| CO_2_ sampling line | REF 8290286, Dräger, Lübeck, Germany | REF 2097307-002, GE, Helsinki, Finland |
| Sodium lime canister | Infinity ID CLIC Absorber 800+, Dräger, Lübeck, Germany | MedisorbTM, Vyaire Medical Oy, Helsinki, Finland |
| CLIC adapter for sodium lime canister | CLIC adapter, Dräger, Lübeck, Germany | - |
| Sevoflurane vaporizer | Vapor 2000 and D-Vapor®, Dräger, Lübeck, Germany | Tec 6 plus and Tec 850 (GE, Freiburg, Germany) |
| Water trap | REF 6872020 Infinity ID WaterLock2, Dräger, Lübeck, Germany | D-fend Pro, GE, Helsinki, Finland |
| Active charcoal filters | Vapor-CleanTM, Dynasthetics LLC, Salt Lake City, UT, USA | Vapor-CleanTM, Dynasthetics LLC, Salt Lake City, UT, USA |
| Breathing circuits and breathing bag | Rüsch Breathing System, REF 191790-210170 Teleflex Medical, Westmeath, Ireland | Rüsch Breathing System, REF 191790-210170 Teleflex Medical, Westmeath, Ireland |
| Bacterial filter | UltiporTM BB25, Pall Medical, Fribourg, Switzerland | UltiporTM BB25, Pall Medical, Fribourg, Switzerland |
| Test lung | SelfTestLungTM, Dräger, Lübeck, Germany | SelfTestLungTM, Dräger, Lübeck, Germany |
